# Chondrocyte activity is increased in psoriatic arthritis and axial spondyloarthritis

**DOI:** 10.1186/s13075-016-1040-z

**Published:** 2016-06-16

**Authors:** Natasja Stæhr Gudmann, Heidi Lausten Munk, Anne Friesgaard Christensen, Leif Ejstrup, Grith Lykke Sørensen, Anne Gitte Loft, Morten Asser Karsdal, Anne-Christine Bay-Jensen, Yi He, Anne Sofie Siebuhr, Peter Junker

**Affiliations:** Nordic Bioscience Biomarkers and Research, Herlev Hovedgade 207, Herlev, 2730 Denmark; Department of Rheumatology, Odense University Hospital, Odense, Denmark; Department of Rheumatology, Vejle Hospital, Vejle, Denmark; Department of Rheumatology, Esbjerg Hospital, Esbjerg, Denmark; Institute of Molecular Medicine, University of Southern Denmark, Odense, Denmark; Department of Rheumatology, Aarhus University Hospital, Aarhus, Denmark

**Keywords:** Spondyloarthritis, Psoriatic arthritis, Type II collagen, Type X collagen, Pro-C2, C-Col10

## Abstract

**Background:**

Psoriatic arthritis (PsA) and axial spondyloarthritis (axSpA) are chronic inflammatory rheumatic diseases with complex origins. Both are characterized by altered extracellular matrix remodeling in joints and entheses that results in destructive and osteochondral proliferative lesions. There is a need for biomarkers reflecting core disease pathways for diagnosis and disease mapping. Pro-C2 reflects mature cartilage collagen type IIB formation, while C-Col10 represents turnover of type X collagen, which is exclusively expressed by hypertrophic chondrocytes. The objectives of this study were to study cartilage metabolism in axSpA and PsA by assessing Pro-C2 and C-Col10 and to evaluate their diagnostic utility against a healthy reference population.

**Methods:**

Patients with PsA (*n* = 101) or axSpA (*n* = 110) were recruited consecutively from three rheumatology outpatient clinics. Demographic and clinical disease measures were recorded. Pro-C2 and C-Col10 were quantified in serum by using newly developed and specific competitive enzyme-linked immunosorbent assays based on monoclonal antibodies. One-way analysis of variance and Tukey’s multiple comparison tests were performed on log-transformed data. ROC curve analysis was carried out to evaluate their discriminative power.

**Results:**

Pro-C2 levels in serum were significantly increased in both axSpA (median concentration 1.11 ng/ml, 0.67–1.64) and PsA (median concentration 1.03 ng/ml, 0.53–1.47) compared with healthy controls (median concentration 0.30 ng/ml, 0.16–0.41) (*p* < 0.0001). Pro-C2 did not differ according to treatment. C-Col10 was slightly but equally elevated in the PsA and axSpA groups vs. the control group, but it was significantly lower in patients with axSpA undergoing tumor necrosis factor-α inhibitor (TNFi) treatment. ROC curve analysis revealed AUCs of 0.85 (95 % CI 0.79–0.89) for axSpA and 0.81 (95 % CI 0.75–0.86) for PsA.

**Conclusions:**

These findings indicate that cartilage collagen metabolism was enhanced in the axSpA and PsA groups compared with the healthy control group. The lower C-Col10 level in patients with axSpA undergoing TNFi treatment may reflect that hypertrophic chondrocytes in axSpA are targeted by TNFi. ROC curve analysis showed a diagnostic potential for Pro-C2 in axSpA and PsA.

## Background

The spondyloarthropathies (SpAs) are chronic inflammatory rheumatic diseases with shared as well as distinctive genetic and clinical features. Joint structural elements and entheses of the axial and peripheral skeleton are principal targets of the diseases [1], which are occasionally also manifested at extraskeletal sites (e.g., anterior uveitis, aortic valvulopathy, and apical lung fibrosis) [1, 2]. Both genetic and environmental factors are implicated in the disease course [3–5], and human leukocyte antigen (HLA)-B27 is overrepresented, occurring in up to 83 % of patients with axial involvement [6].

Disease presentation and progression in SpAs differ between individuals. If left untreated, many patients will experience spinal and peripheral joint deformities due to altered bone and cartilage turnover. Osteochondral proliferative lesions are hallmarks of axial and entheseal involvement [7]. Current treatments include nonsteroidal anti-inflammatory drugs, methotrexate, and biological agents such as tumor necrosis factor-α inhibitors (TNFi) [8]. Early intervention is recommended to prevent or slow the progression rate. However, early diagnosis is challenging because of a lack of pathognomonic features and/or laboratory tests, and the average diagnostic delay has been reported to be approximately 8–11 years [7]. In psoriatic arthritis (PsA), early diagnosis has been shown to significantly decrease morbidity and increase quality of life [9]. Thus, interest in developing sensitive and specific tools for diagnostic and prognostic purposes is increasing. The utility of several cartilage and bone biochemical markers has been assessed, particularly in osteoarthritis and rheumatoid arthritis, and also, although less intensively, in axial spondyloarthritis (axSpA) and PsA [10]. Thus, markers of cartilage formation (type II collagen [CII] [11]) and degradation (collagen type II cleavage [C2C] [11] and C-terminal telopeptide of type II collagen [CTX-II] [12]) have been shown to be elevated in axSpA and PsA. Similar findings have been reported recently with use of the regenerative collagen type IIA N-terminal propeptide (PIIANP) and matrix metalloproteinase-generated type II collagen degradation fragment (C2M) [13]. The Pro-C2 assay is the first laboratory tool for specific measurement of the N-terminal propeptide of the procollagen IIB splice variant (PIIBNP), which is the most prevalent collagen species in healthy adult cartilage. By contrast, previous candidate markers for type II collagen formation either do not segregate other variants such as CII [11], NPII [14], and PIINP [15] or are specific for PIIANP, which is expressed mainly during embryogenesis, fracture healing, and cartilage regeneration [16–18]. Additionally, C-Col10 is, to our knowledge, the first marker available for the assessment of type X collagen turnover, and so far it has been applied only to patients with osteoarthritis [19].

On the basis of these observations, we hypothesized that remodeling of mature collagen IIB and collagen X, a minor collagen species that is produced only by hypertrophic chondrocytes, is increased in axSpA and PsA. The aims of this study were (1) to study cartilage collagen metabolism in further detail using novel molecular markers for the synthesis and turnover of mature collagen IIB (Pro-C2) [20] and collagen X (C-Col10) [19] in well-characterized cohorts of patients with axSpA and PsA, and (2) to assess their diagnostic utility in comparison with healthy control subjects.

## Methods

### Study populations

Patients from three rheumatology centers in Southern Denmark were recruited from November 2011 through February 2014 (33–120 patients per center). The inclusion criteria and patient characteristics have previously been published [13]. In short, 110 patients with axSpA classified according to the Assessment of SpondyloArthritis international Society (ASAS) criteria [21, 22] and 101 patients with PsA fulfilling the Classification of Psoriatic Arthritis (CASPAR) criteria [23] were enrolled in the study. Sixteen of the patients fulfilled both the CASPAR and the ASAS criteria, and these patients were labeled as having PsA in this study. Exclusion criteria were age <18 years and age >75 years, past or present malignancy (except nonmalignant skin cancer), congestive cardiac disease (New York Heart Association classes III and IV), pulmonary disease (dyspnea at rest), serum creatinine above the upper limit of normal, serum concentration of alanine aminotransferase more than two times the upper limit of normal, infection with hepatitis B or hepatitis C virus, and/or presence of other chronic inflammatory diseases. The patient characteristics recorded included sex, body mass index (BMI), age, smoking habit, alcohol consumption, treatment, and HLA-B27 status. Disease measures recorded included 68 swollen joint count (68SJC), 68 tender joint count (68TJC), Spondyloarthritis Research Consortium of Canada enthesitis index, Health Assessment Questionnaire (HAQ), visual analogue scale (VAS; patient pain, patient global, patient fatigue, and physician global scores on a 0–10 scale), Bath Ankylosing Spondylitis Disease Activity Index (BASDAI), Disease Activity Score in 28 joints (DAS28), and Ankylosing Spondylitis Disease Activity Score (ASDAS). X-rays or magnetic resonance imaging (MRI) scans of sacroiliac joints of patients with axSpA and of patients with PsA with axial involvement were available when the disease was diagnosed, but they were not repeated in this study. Twelve patients with axSpA had ankylosis of the sacroiliac joints visualized on x-rays or MRI scans. Demographic data and disease parameters are summarized in Table 1. One hundred eighteen blood donors aged 20–65 years served as healthy control subjects. Blood samples were collected in plain tubes from subjects in nonfasting state. The samples were allowed to clot for 30–120 minutes and then centrifuged for 12 minutes at 2200 rpm. Serum samples were frozen at −80 °C until used for analysis.

**Table 1:** Patient characteristics, disease measures, and serum levels of Pro-C2 and C-Col10

	axSpA (*n* = 110)	PsA (*n* = 101)	Control
Male sex, %	72 %	59 %	
Age, years	36.6 (35.3–38.0)	37.0 (35.6–38.5)	
BMI, kg/m^2^	25.5 (24.8–26.3)	27.4 (26.2–28.5)	
Smoker, %	36 %	35 %	
HLA-B27 positive, %	87 %	20 %	
Disease duration, years	6.4 (5.4–7.5)	6.7 (5.6–7.8)	
VAS			
Patient global	34 (29–39)	39 (34–45)	
Patient pain	32 (27–37)	32 (27–37)	
Patient fatigue	40 (34–45)	43 (37–49)	
Physician global	4 [1–16]	3 [1–14]	
BASDAI	31 (26–35)	36 (31–41)	
BASFI	23 (19–27)	27 (22–32)	
BASMI	10 [0–20]	10 [0–10]	
ASDAS (CRP)	2.0 (1.8–2.3)	2.1 (1.9–2.4)	
Swollen joints, %	9 %	38 %	
hs-CRP, mg/dl	3 [1–7]	3 [1–9]	
Pro-C2, ng/ml	1.11 [0.67-1.64]	1.03 [0.53-1.47]	0.30 [0.16-0.41]
Col-X, ng/ml	0.45 [0.28-0.67]	0.42 [0.31-0.72]	0.33 [0.31-0.45]

*Abbreviations: ASDAS* Ankylosing Spondylitis Disease Activity Score, *axSpA* axial spondyloarthritis, *BASDAI* Bath Ankylosing Spondylitis Disease Activity Index, *BASFI* Bath Ankylosing Spondylitis Functional Index, *BASMI* Bath Ankylosing Spondylitis Metrology Index, *BMI* body mass index, *HLA-B27* human leukocyte antigen B27, *hs-CRP* high-sensitivity C-reactive protein, *PsA* psoriatic arthritis, *VAS* visual analogue scale

Data are presented as mean (95 % CI) for variables with a normal distribution and as median [25th–75th percentile range] for variables that were not normally distributed

### Biomarker measurements

The Pro-C2 competitive enzyme-linked immunosorbent assay (ELISA) has been described previously by Gudmann et al. [20]. In short, 96-well streptavidin-coated plates (Roche Diagnostics, Risch-Rotkreuz, Switzerland) were coated with the biotinylated synthetic peptide Bio-QDVRQPG, dissolved in assay buffer (100 mM PBS, 1 % bovine serum albumin, 0.1 % Tween-20, 0.36 % 5-bromo-5-nitro-1,3-dioxane [Bronidox; BASF, Florham Park, NJ, USA], 8 % NaCl, pH 7.4), and incubated for 30 minutes at 20 °C. The peptide calibrator or sample in a quantity of 20 μl was added to appropriate wells, followed by 100 μl of antibody of the NB443-3-2-1 clone. The plates were then incubated for 2 h. Afterward, 100 μl of EnVision (Dako, Glostrup, Denmark) was added, and the plate was incubated for 1 h. Finally, 100 μl of 3,3′,5,5′-tetramethylbenzidine (TMB) (Kem-En-Tec Nordic, Taastrup, Denmark) was added, and the plate was incubated for 15 minutes at 20 °C in the dark. The C-Col10 ELISA method has been described in detail previously by He et al. [19]. Measurements were performed as follows: 100 μl of biotinylated peptide of the sequence SFSGFLVAPM was added to plates precoated with streptavidin (Roche Diagnostics) and incubated at 20 °C for 30 minutes. Standard or patient samples in a quantity of 20 μl was then added to the appropriate wells together with 100 μl of peroxidase-labeled antibody NB509-11G8 and incubated at 4 °C overnight with shaking. Afterward, 100 μl of TMB was added to each well of the plates and incubated in the dark at 20 °C for 15 minutes. All samples were measured in duplicate, and incubation steps included shaking at 300 rpm for both assays. After each incubation except the last one in which TMB was added, the plates were washed five times in washing buffer (20 mM Tris, 50 mM NaCl, pH 7.2). The TMB reaction was stopped by adding 100 μl of stopping solution (1 % H_2_SO_4_) and measured at 450 nm with 650 nm as the reference.

### Statistics

All analyses were performed using MedCalc statistical software version 14.8.1 (2014 release; MedCalc Software bvba, Ostend, Belgium). Means were calculated for variables with a normal distribution, and medians were calculated for variables that were not normally distributed. Biomarker levels and parameters that did not meet the criteria for normal distribution were log-transformed. Normally distributed variables were compared using *t* tests, and one-way analysis of variance followed by Tukey’s multiple comparisons test were applied for intergroup comparisons. Correlation analyses were performed by using Pearson’s test. A *p* value ≤0.05 was considered statically significant. The discriminative power of the serum markers between healthy and diseased states was calculated by using ROC and expressed by the AUC. The Youden index was applied to determine the optimum sensitivity and specificity and the corresponding cutoff values.

## Results

### Patient demographics

Patient demographics and disease characteristics are presented in Table 1. Male sex was more common in both axSpA (72 %) and PsA (59 %) than in the control group (50 %). The clinical parameters are listed for patients, but they were not available for healthy blood donors.

### Cartilage turnover is increased in axSpA and PsA

**Fig. 1:**
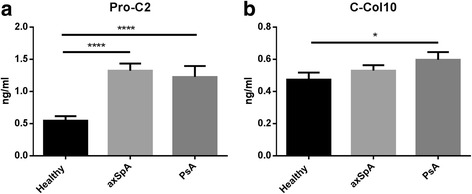
**a** Pro-C2 was significantly increased in the axial spondyloarthritis (axSpA) and psoriatic arthritis (PsA) groups compared with the healthy control group. **b** C-Col 10 concentrations were slightly but significantly higher in the PsA group compared with the healthy control group. Error bars are shown as SEM. One-way analysis of variance was performed for intergroup comparisons. *p < 0.05, ****p < 0.0001

Pro-C2 was significantly increased in the axSpA and PsA groups compared with the healthy control group (Fig. 1a). Similarly, C-Col10 was significantly elevated in patients with PsA compared with healthy control subjects, and the same trend was observed in patients with axSpA (Fig. 1b). Of note, in patients with axSpA naïve to TNFi treatment (*n* = 34), C-Col10 was significantly higher in patients with axSpA than in TNFi-treated patients and healthy control subjects (*p* = 0.008). There was no significant difference in Pro-C2 or C-Col10 between the axSpA and PsA groups.

### Pro-C2 associations

Pro-C2 correlated with ASDAS in the patients with axSpA naïve to disease-modifying antirheumatic drugs (DMARDs). In treatment-naïve patients with PsA Pro-C2, there was a borderline correlation with age, BMI, and disease duration. Patients with axSpA currently being treated with synthetic and/or biological DMARDs had a significant correlation between Pro-C2 and HAQ and Bath Ankylosing Spondylitis Functional Index (BASFI). HAQ and Pro-C2 were also significantly correlated in currently treated patients with PsA (Table 2).

**Table 2:** Pearson correlations between Pro-C2 levels in axial spondyloarthritis and psoriatic arthritis and clinical parameters

	axSpA				PsA			
	Total (*n* = 110)	Treatment-naïve (*n* = 34)	Currently treated, total (*n* = 63)	Previously treated (*n* = 13)	Total (*n* = 101)	Treatment-naïve (*n* = 13)	Currently treated, total (*n* = 76)	Previously treated (*n* = 12)
Age	ns	ns	ns	ns	ns	*r* = −0.55	ns	ns
						*p* = −0.051		
BMI	ns	ns	ns	ns	ns	*r* = 0.60	ns	ns
						*p* = 0.032		
Disease duration	ns	ns	ns	*r* = 0.54	ns	*r* = 0.55	ns	ns
				*p* = 0.030		*p* = 0.052		
ASDAS	ns	*r* = 0.45	ns	ns	ns	ns	ns	ns
		*p* = 0.008						
HAQ	ns	ns	*r* = 0.47	ns	*r* = −0.34	ns	*r* = −0.35	ns
			*p* = 0.010		*p* = 0.004		*p* = 0.008	
Sex	ns	ns	ns	ns	ns	ns	ns	ns
BASDAI	ns	ns	ns	ns	ns	ns	ns	ns
BASFI	ns	ns	*r* = −0.30	ns	ns	ns	ns	ns
			*p* = 0.008					
SJC68	ns	ns	ns	ns	ns	ns	ns	*r* = −0.92
								*p* = 0.0001
TJC68	ns	ns	ns	ns	ns	ns	ns	*r* = −0.89
								*p* = 0.0003

*Abbreviations: ASDAS* Ankylosing Spondylitis Disease Activity Score, *axSpA* axial spondyloarthritis, *BASDAI* Bath Ankylosing Spondylitis Disease Activity Index, *BASFI* Bath Ankylosing Spondylitis Functional Index, *BASMI* Bath Ankylosing Spondylitis Metrology Index, *BMI* body mass index, *HAQ* Health Assessment Questionnaire, *ns* not significant, *SJC68* 68 swollen joint count, *TJC68* 68 tender joint count

**Fig. 2:**
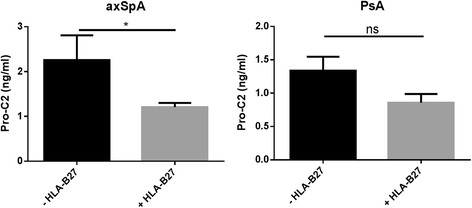
Pro-C2 was significantly lower in patients with axial spondyloarthritis (axSpA) who were positive for the human leukocyte antigen (HLA)-B27 gene compared with the HLA-B27-negative patients. In patients with psoriatic arthritis (PsA), Pro-C2 did not differ significantly according to HLA-B27 status (*p*=0.31). Error bars are shown as SEM. Student’s *t* test was applied to compare the two groups. *p < 0.05. *ns* not significant

HLA-B27-positive patients with axSpA had significantly lower Pro-C2 levels than HLA-B27-negative patients (*p* = 0.026) (Fig. 2). In contrast, we found no statistically significant differences in Pro-C2 levels when we compared subjects according to smoking status (*p* = 0.51), male vs. female sex (*p* = 0.46), current treatment (neither biologics [*p* = 0.50] nor synthetic DMARDs [*p* = 0.15]), SJC28 (*p* = 0.63), or TJC28 (*p* = 0.28). We observed no significant differences in Pro-C2 when we compared subjects according to HLA-B27 status in PsA (*p* = 0.18), and this applied to other clinical parameters as well (data not shown).

### C-Col10 associations

In patients with axSpA who were naïve to treatment, a weak correlation was found between C-Col10 and disease duration and SJC68. In patients with axSpA currently receiving treatment (synthetic and/or biologic DMARDs), a correlation was found between sex and C-Col10. No correlation was found between type X collagen in serum and disease parameters in patients with PsA (Table 3).

**Table 3:** Pearson correlations between C-Col10 levels in axial spondyloarthritis and psoriatic arthritis and clinical parameters

	axSpA				PsA			
	Total (*n* = 110)	Treatment-naïve (*n* = 34)	Currently treated (*n* = 63)	Previously treated (*n* = 13)	Total (*n* = 101)	Treatment-naïve (*n* = 13)	Currently treated (*n* = 76)	Previously treated (*n* = 12)
Age	ns	ns	ns	ns	ns	ns	ns	ns
BMI	ns	ns	ns	ns	ns	ns	ns	ns
Disease duration	ns	*r* = −0.38	ns	ns	ns	ns	ns	ns
		*p* = 0.027						
ASDAS	ns	ns	ns	ns	ns	ns	ns	ns
Alcohol consumption	ns	ns	ns	*r* = −0.60	ns	ns	ns	ns
				*p* = 0.022				
HAQ	ns	ns	ns	ns	ns	ns	ns	ns
Sex	ns	ns	*r* = 0.30	ns	ns	ns	ns	ns
			*p* = 0.021					
BASDAI	ns	ns	ns	ns	ns	ns	ns	ns
SJC68	ns	*r* = −0.36	ns	ns	ns	ns	ns	ns
		*p* = −0.037						
TJC68	*r* = −0.20	ns	ns	ns	ns	ns	ns	*r* = −0.73
	*p* = 0.043							*p* = 0.011

*Abbreviations: ASDAS* Ankylosing Spondylitis Disease Activity Score, *axSpA* axial spondyloarthritis, *BASDAI* Bath Ankylosing Spondylitis Disease Activity Index, *BMI* body mass index, *HAQ* Health Assessment Questionnaire, *ns* not significant, *PsA* psoriatic arthritis, *SJC68* 68 swollen joint count, *TJC68* 68 tender joint count

**Fig. 3:**
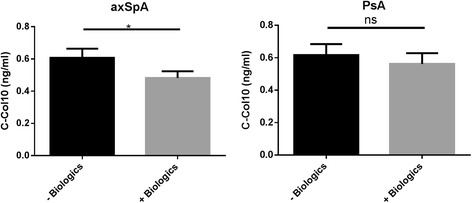
C-Col10 was significantly higher in patients with axial spondyloarthritis (axSpA) naïve to biological treatment than in those currently or formerly treated with biologic DMARDs. The same trend was observed in patients with psoriatic arthritis (PsA), though it did not reach statistical significance. Error bars are shown as SEM. Student’s *t* test was applied to compare the two groups. *p < 0.05. *ns* not significant

C-Col10 levels were significantly lower in patients with axSpA who received or had previously received DMARD biologics (*p* = 0.019) than in those who did not, whereas treatment with a synthetic DMARD did not affect C-Col10 concentration (*p* = 0.96) (Fig. 3). This pattern did not apply to patients with PsA (*p* = 0.34). C-Col10 did not associate with sex, HLA-B27, SJC28, TJC28, or disease state as assessed using either DAS28, ASDAS, BASDAI, or BASFI in patients with axSpA or PsA (data not shown).

### Discriminative power of Pro-C2 and C-Col10

**Fig. 4:**
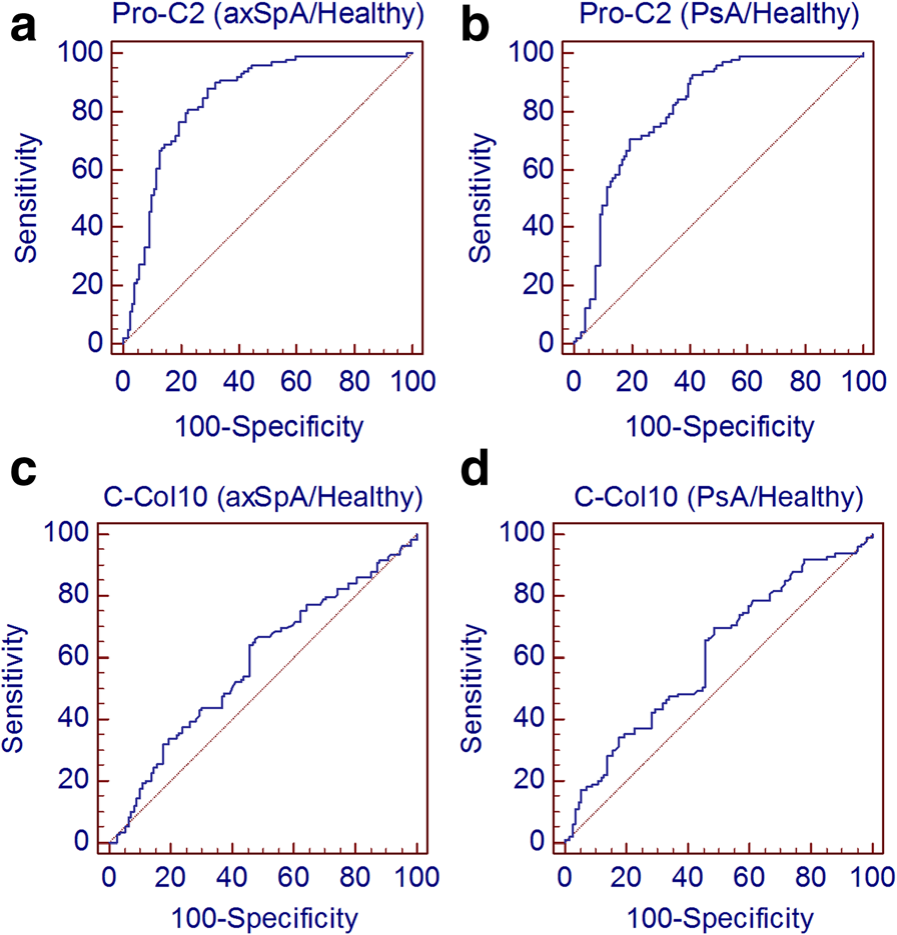
ROC analysis of (**a**) Pro-C2 in the axial spondyloarthritis (axSpA) group vs. the healthy control group, (**b**) Pro-C2 in the psoriatic arthritis (PsA) group vs. the healthy control group, (**c**) C-Col10 levels in the axSpA group vs. the healthy control group, and (**d**) C-Col10 in the PsA group vs. the healthy control group

The discriminating power between the healthy control, axSpA, and PsA groups was assessed by ROC analysis (Fig. 4). The AUCs of the healthy control group vs. disease groups were, for both biomarkers, approximately the same for the two diseases. For Pro-C2, the healthy control group vs. the axSpA group had an AUC of 0.85 (95 % CI 0.79–0.89); for the healthy control group vs. the PsA group, the AUC was 0.81 (95 % CI 0.75–0.86) (see Table 4). For C-Col10, the AUC of the healthy control group vs. the disease groups was lower than that for Pro-C2 but approximately the same in the two disease groups. The AUC for the healthy control group vs. the axSpA group was 0.58 (95 % CI 0.51–0.65), and for the healthy control group vs. the PsA group it was 0.61 (95 % CI 0.54–0.67) (Table 4).

**Table 4:** ROC analysis of ability of Pro-C2 and C-Col10 to differentiate between healthy control, psoriatic arthritis, and axial spondyloarthritis groups

	AUC (95 % CI)	Youden index	Sensitivity	Specificity
Pro-C2				
Healthy vs. axSpA	0.85 (0.79–0.89)	0.58	87.9	70.6
Healthy vs. PsA	0.81 (0.75–0.86)	0.51	92.7	58.8
axSpA vs. PsA	0.54 (0.47–0.61)	0.13	17.43	95.2
C-Col10				
Healthy vs. axSpA	0.58 (0.51–0.65)	0.18	67.0	51.4
Healthy vs. PsA	0.61 (0.54–0.67)	0.21	69.7	51.4
axSpA vs. PsA	0.52 (0.45–0.59)	0.11	94.5	16.8

*axSpA* axial spondyloarthritis, *PsA* psoriatic arthritis

## Discussion

Structural elements in joints and entheses are major disease targets in axSpA [24] and PsA [25]. Therefore, in the present study, we measured two recently developed cartilage biochemical markers—C-Col10 and Pro-C2—to study chondrocyte differentiation and mature cartilage collagen IIB formation in axSpA and PsA.

Pro-C2 concentrations were significantly elevated in both the axSpA and PsA groups compared with levels in control subjects. In addition, C-Col10 levels were slightly higher in both disease subsets than the levels in control subjects, although they reached statistical significance only in the PsA group. Pro-C2 correlated with ASDAS in untreated patients with axSpA and with HAQ and BASFI in TNFi-treated patients with axSpA. C-Col10 was weakly associated with SJC68. While Pro-C2 was not influenced by TNFi treatment, C-Col10 was lower in patients with axSpA currently or previously receiving biologics than in patients naïve to TNFi treatment. Good discrimination for Pro-C2 was seen for patients with axSpA and patients with PsA vs. healthy subjects.

Guidelines for treatment of axSpA and PsA rely mainly on clinical assessment of disease activity and progression [26, 27]. Thus, biologically plausible biomarkers may provide additional information regarding diagnosis and assessment of disease activity, and they may also help to guide treatment [28, 29]. We previously reported that type II collagen seromarkers differ between axSpA and PsA [13]. However, this report was based on the marker of mature collagen II (C2M) [30] and a marker of regenerative collagen II formation (PIIANP) [31]. In the present study, we wanted to extend these previous observations using Pro-C2, a marker of mature collagen synthesis, and C-Col10, a fragment of collagen X. Thus, Pro-C2 and PIIANP reflect the synthesis of two different splice variants of procollagen II. Type IIA procollagen is expressed mainly during embryogenesis [32] and has been found to be reexpressed in osteoarthritis [16]. The PIIANP splice variant is considered to represent a dedifferentiated version of type II collagen [31]. By contrast, collagen type IIB N-terminal propeptide (PIIBNP) is considered to be the only procollagen expressed during type II collagen formation in healthy adult cartilage [17]. Pro-C2 is specific for evaluation of the PIIBNP splice variant [20]. This allows for discrimination between structural type IIB vs. regenerative cartilage type IIA formation in healthy and diseased states. The present study shows that mature collagen II synthesis as reflected by Pro-C2 is increased in both axSpA and PsA, while our previous study showed that PIIANP was increased only in currently untreated patients with PsA and in treatment-naïve patients with axSpA compared with control subjects [13]. Notably, Pro-C2 was not affected by DMARD treatment in axSpA or PsA, while PIIANP was lower in treated than in untreated patients, probably reflecting suppression of a cartilage pathogenic pathway [13]. The increased on-treatment Pro-C2 levels likely reflect that mature cartilage collagen IIB synthesis is maintained despite drug treatment. The increased Pro-C2 levels correlated significantly with physical function scores but not with joint counts, which may reflect a mature chondrocyte response to altered mechanical influences on cartilage elicited by synovial and entheseal pathologies [33]. The lower Pro-C2 concentration in HLA-B27-positive patients with axSpA than in HLA-B27-negative patients suggests that the HLA-B27 gene is implicated in the regulation of type II collagen expression, as recently proposed for procollagen IIA in axSpA [13]. Our findings add to those described by Pedersen et al., who reported that the urinary excretion of CTX-II, a marker of collagen II degradation, was increased in axSpA but decreased during TNFi treatment [12]. Thus, the current increased Pro-C2 level may reflect an anabolic chondrocyte response to counterbalance increased collagen type II degradation in active disease.

By analogy with osteoarthritis, we hypothesized that C-Col10 levels would be increased in SpA as a marker of syndesmophyte outgrowth similar to osteophyte formation [21, 22, 34, 35]. Thus, C-Col10 was significantly increased in patients with PsA as compared with control subjects. The same trend was observed in axSpA, although it did not reach statistical significance. However, C-Col10 was significantly lower in patients with axSpA treated with biologic DMARDs than in those naïve to biological treatment, indicating that hypertrophic chondrocytes and collagen X expression are suppressed by TNFi. This response contrasts with Pro-C2, indicating that household collagen II formation is left unaffected by TNF-α inhibition, while potential cartilage osteochondral precursor collagen is suppressed. This observation and the association between C-Col10 and SJC lend support to the concept that chondrocyte hypertrophy and increased expression of type X collagen are implicated in the axSpA disease pathway. However, in a recent study, Bleil et al. [23] reported that chondrocyte hypertrophy was not present in the facet joints of patients with axSpA as assessed by immunohistochemistry. These authors studied cartilage from patients with advanced ankylosing spondylitis, in which the chondrocyte phenotype and distribution may be different from early active disease and therefore may have a different type X collagen expression profile. C-Col10 levels in osteoarthritis (OA) have previously been studied in patients stratified according to the Kellgren and Lawrence scoring system. In that study, C-Col10 was significantly elevated in patients with intermediate structural OA changes vs. patients with severe or no abnormalities [19]. However, the cartilage pathology of OA may differ from that of axSpA, and the patient populations were not comparable in terms of age, sex, or disease duration. Alternatively, the increased release of type X collagen fragments may originate from cartilage in areas other than the facet joints in patients with axSpA. However, the risk of concurrent osteoarthritis in the present patient populations is considered to be low because the median age was only around 35 years.

Some limitations of the present study should be considered. The cross-sectional design provides an opportunity to evaluate the biochemical disease profile at a specific time point, but it does not provide prognostic information. No updated x-rays or MRI scans were available. Since this is the first report on Pro-C2 in a clinical setting, it is uncertain how the present results compare with findings in other joint diseases. The strengths of this study are inclusion of two large and clinically well-characterized patient populations with axSpA and PsA and a large number of healthy control subjects. In addition, this is the first time that Pro-C2 and C-Col10 have been applied to axSpA and PsA.

## Conclusions

This study presents a biochemical cartilage marker profile in two disease subsets within the spondyloarthropathy complex indicating the presence of hypertrophic and enhanced chondrocyte activity, particularly in active disease. While increased collagen IIB production was unaffected by TNFi treatment, type X collagen of hypertrophic chondrocytes was suppressed by these agents. This may indicate that the SpA-related type II collagen response of normal chondrocytes is preserved, while the collagen type X expression of cartilaginous and enthesopathic lesions is modified by TNFi. Prospective studies are needed to validate this concept.

## Abbreviations

ASAS, Assessment of SpondyloArthritis international Society; ASDAS, Ankylosing Spondylitis Disease Activity Score; axSpA, axial spondyloarthritis; BASDAI, Bath Ankylosing Spondylitis Disease Activity Index; BASFI, Bath Ankylosing Spondylitis Functional Index; BASMI, Bath Ankylosing Spondylitis Metrology Index; BMI, body mass index; CASPAR, Classification of Psoriatic Arthritis; C2C, collagen type II cleavage; C2M, matrix metalloproteinase-generated type II collagen degradation fragment; CII, type II collagen; CRP, C-reactive protein; CTX-II, C-terminal telopeptide of type II collagen; DAS28, Disease Activity Score in 28 joints; DMARD, disease-modifying antirheumatic drug; ELISA, enzyme-linked immunosorbent assay; HAQ, Health Assessment Questionnaire; HLA-B27, human leukocyte antigen B27; hs-CRP, high-sensitivity C-reactive protein; MRI, magnetic resonance imaging; NPII, N-terminal procollagen type II; NPIIB, N-terminal propeptide of the procollagen IIB splice variant; ns, not significant; OA, osteoarthritis; PIIANP, collagen type IIA N-terminal propeptide; PIIBNP, collagen type IIB N-terminal propeptide; PIINP, collagen type II N-terminal propeptide; PsA, psoriatic arthritis; SJC68, 68 swollen joint count; SpA, spondyloarthropathy; TJC68, 68 tender joint count; TMB, 3,3′,5,5′-tetramethylbenzinidine; TNFi, tumor necrosis factor-α inhibitor; VAS, visual analogue scale
